# Supplementary material for: Effects of Host-Adaptive Mutations on Hop Stunt Viroid Pathogenicity and Small RNA Biogenesis
Source: Int J Mol Sci. 2020 Oct 6;21(19):7383. doi: 10.3390/ijms21197383 (PMC7582576; doi:10.3390/ijms21197383)
Supplement: Supplementary file 1 [file ijms-21-07383-s001.zip › Supplementary files/Supplementary figures1-5.docx]

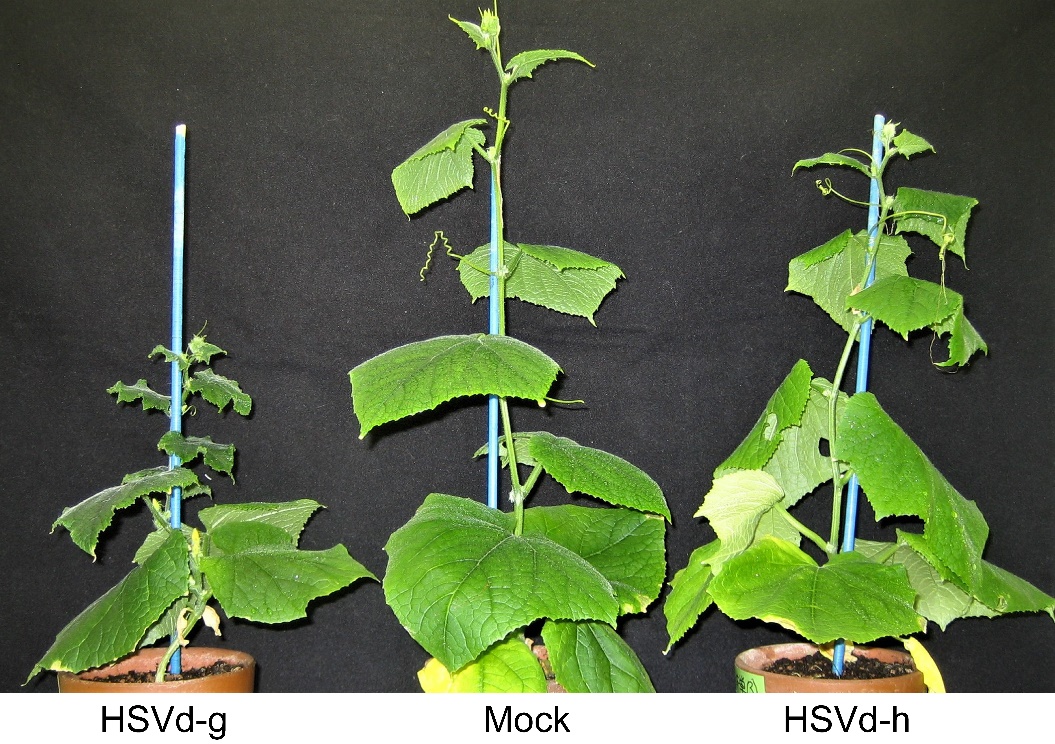


**Supplementary Figure 1**. Symptoms of cucumber plants infected by HSVd-g and HSVd-h at 14 days post inoculation (dpi).


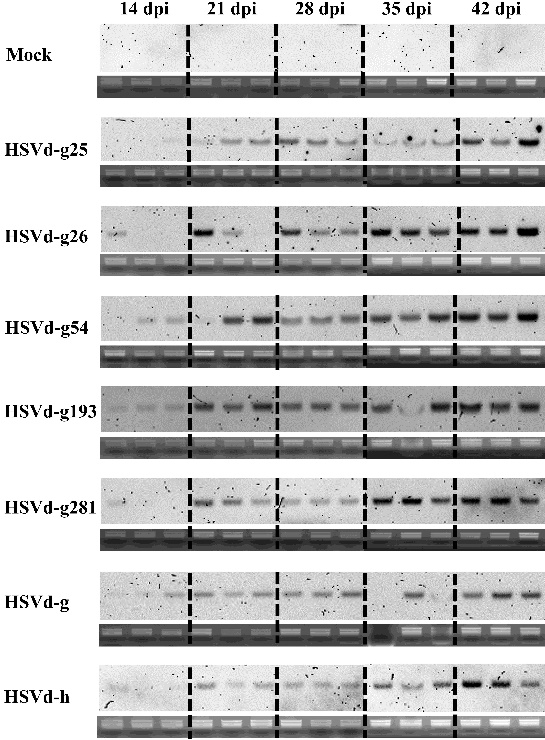


**Supplementary Figure 2**. Northern-blot analysis of the cucumber plants individually inoculated by HSVd-h, HSVd-g, and five mutants of HSVd-g (HSVd-g25,-g26,-g54,-g193,and -g281). Three out five inoculated plants were randomly selected for detection.


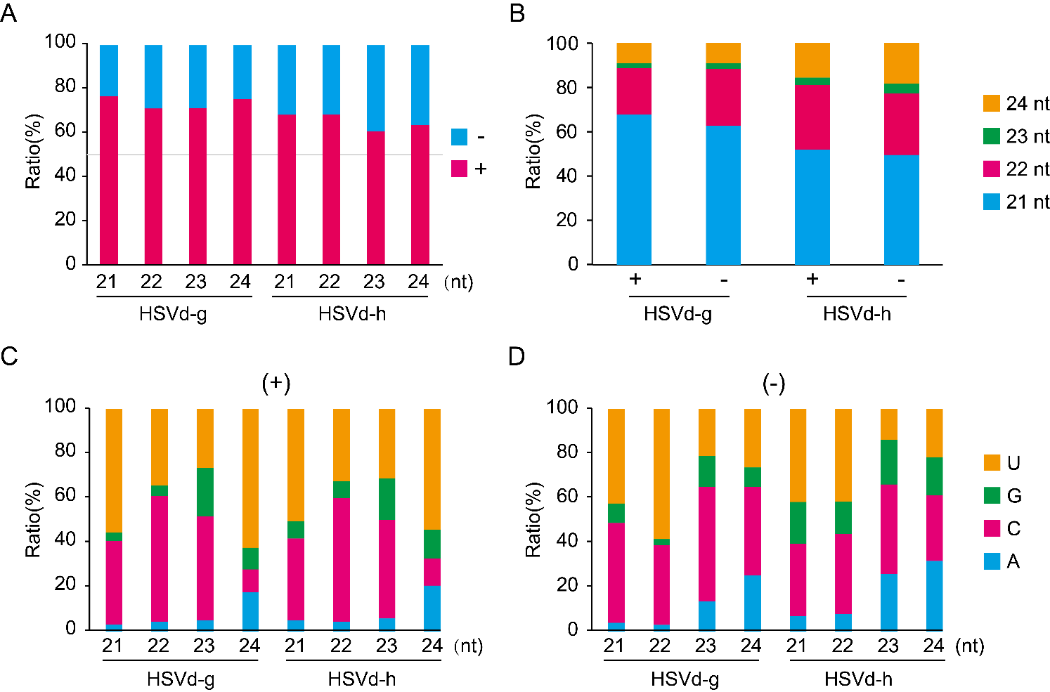


**Supplementary Figure 3**. Characteristics of HSVd-sRNA derived from cucumber plants infected with either HSVd-g or HSVd-h.

**
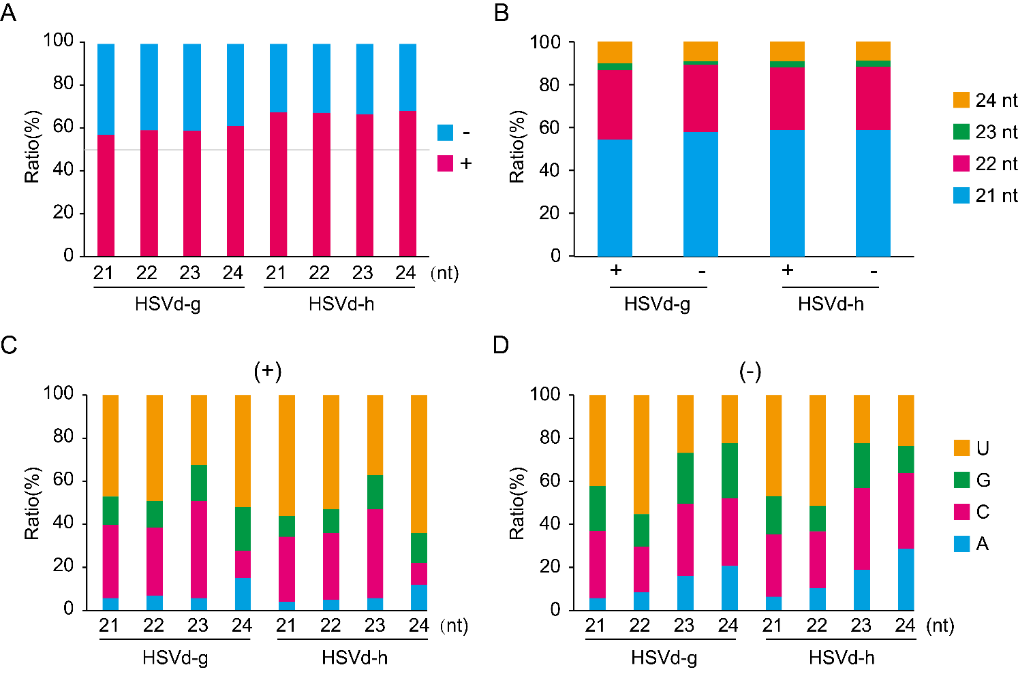
**

**Supplementary Figure 4.** Characteristics of HSVd-sRNA derived from hop plants infected with either HSVd-g or HSVd-h.

**
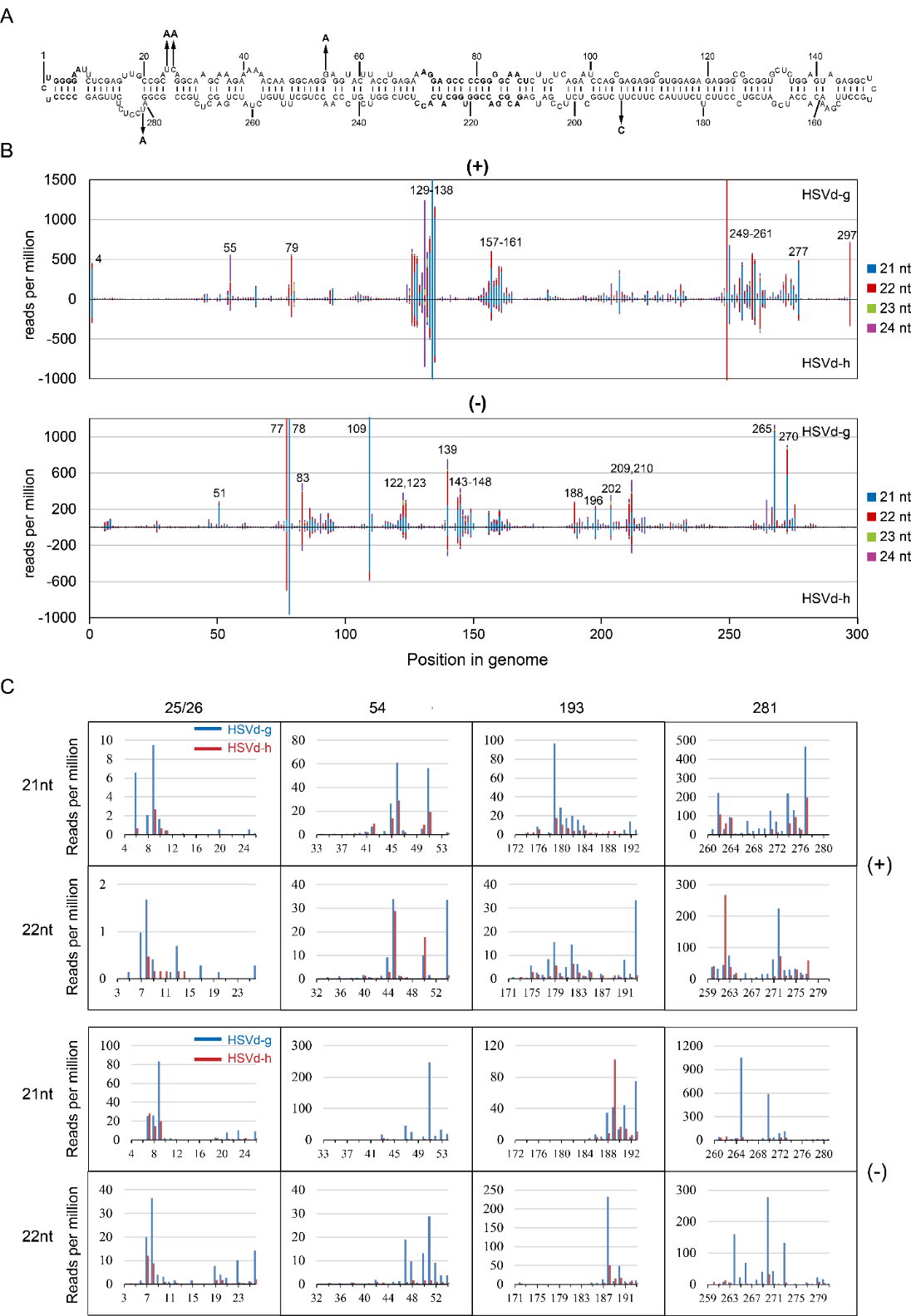
**

**Supplementary Figure 5.** Sequence profiles of HSVd-sRNAs derived from the genomic (+) and anti-genomic (−)-strands of HSVd-g or HSVd-h in hop. The top panel **(A)** shows the predicted secondary structure of HSVd-g. The five hop-adaptive mutations between HSVd-g and HSVd-h are indicated by the arrows. sRNA distribution profiles shown in panel **(B)** represent the sum of 21-24-nt HSVd-sRNA populations recovered from cucumber plants infected with HSVd-h or HSVd-g plotted along the linearized HSVd genome (nucleotides 1-297 from left to right). sRNA distribution profiles of HSVd-g and HSVd-h are compared for genomic (upper panel) and anti-genomic (lower panel) strands. Genome positions of selected hot-spot peaks are marked by corresponding numbers. Arrowheads indicate sRNAs containing adaptive mutations that distinguish HSVd-g from HSVd-h and accumulate to obviously different levels. Details of their amounts and sequences are shown in panel **(C)**.
