# Supplementary material for: Effects of Host-Adaptive Mutations on Hop Stunt Viroid Pathogenicity and Small RNA Biogenesis
Source: Int J Mol Sci. 2020 Oct 6;21(19):7383. doi: 10.3390/ijms21197383 (PMC7582576; doi:10.3390/ijms21197383)
Supplement: Supplementary file 1 [file ijms-21-07383-s001.zip › Supplementary files/Table S1 and S2.docx]

**Supplementary Table 1.** Overall features of HSVd-sRNA isolated from cucumber and hop plants infected with HSVd-h or HSVd-g (the 1^st^ sRNA analysis).

| Host/  Time | Variant | Clean reads  (Million) | HSVd-sRNA reads (21-24 nt) | | | HSVd-sRNA  level (%) | Ratio (+/-) |
| --- | --- | --- | --- | --- | --- | --- | --- |
|  |  |  | Total | + | - |  |  |
| Cucumber/  4 wpi | HSVd-h | 6.82 | 83,808 | 55,464 | 28,344 | 1.23 | 1.94 |
|  | HSVd-g | 7.07 | 103,546 | 77,050 | 26,496 | 1.46 | 2.91 |
| Hop/  8 wpi | HSVd-h | 6.34 | 194,244 | 129,234 | 65,010 | 3.07 | 1.99 |
|  | HSVd-g | 7.16 | 427,145 | 245,266 | 181,879 | 5.97 | 1.35 |

**Supplementary Table 2.** Overall features of HSVd-sRNA isolated from cucumber plants infected with HSVd-h and HSVd-g54 at 14 dpi and 28 dpi (the 2^nd^ sRNA analysis).

| **Time** | **Variant** | **Clean reads**  **(Million)** | **HSVd-sRNA reads (21-24 nt)** | | | HSVd-sRNA  level (%) | Ratio (+/-) |
| --- | --- | --- | --- | --- | --- | --- | --- |
|  |  |  | **Total** | + | - |  |  |
| 14 dpi | HSVd-h | 13.93 | 8,551 | 5,348 | 3,167 | 0.06 | 1.69 |
|  | HSVd-g54 | 12.09 | 17,258 | 11,162 | 6,096 | 0.14 | 1.83 |
| 28 dpi | HSVd-h | 10.02 | 63,319 | 40,745 | 22,574 | 0.63 | 1.80 |
|  | HSVd-g54 | 12.29 | 97,074 | 66,304 | 30,770 | 0.79 | 2.15 |
